# Feasibility and outcomes of a multi-function mobile health approach for the schizophrenia spectrum: App4Independence (A4i)

**DOI:** 10.1371/journal.pone.0219491

**Published:** 2019-07-15

**Authors:** Sean A. Kidd, Laura Feldcamp, Amos Adler, Linda Kaleis, Wei Wang, Klara Vichnevetski, Kwame McKenzie, Aristotle Voineskos

**Affiliations:** 1 University of Toronto, Department of Psychiatry, Toronto, Canada; 2 Centre for Addiction and Mental Health, Toronto, Canada; 3 MEMOTEXT, Toronto, Ontario, Canada; University of California Los Angeles, UNITED STATES

## Abstract

Relative to the large investments in mobile health (mHealth) strategies for mental illnesses such as anxiety and depression, the development of technology to facilitate illness self-management for people with schizophrenia spectrum illnesses is limited. This situation falls out of step with the opportunity mHealth represents for providing inexpensive and accessible self-care resources and the routine use of mobile technologies by people with schizophrenia. Accordingly, the focus of this study was upon the feasibility of a schizophrenia-focused mobile application: App4Independence (A4i). A4i is a multi-feature app that uses feed, scheduling, and text-based functions co-designed with service users to enhance illness self-management. This study was completed in a large urban Canadian centre and employed pre-post assessments over a 1-month period that examined medication adherence, personal recovery, and psychiatric symptomatology. App use metrics were assessed as was qualitative feedback through semi-structured interview. Findings are reported in line with the World Health Organization mHealth Evidence and Assessment (mERA) checklist. Among the 38 individuals with a primary psychosis who participated, there was no research attrition and classic retention on the app was 52.5%. Significant improvement was observed in some psychiatric symptom domains with small-medium effects. Significant change in recovery engagement and medication adherence were not observed after controlling for multiple comparisons. Those who interacted with the app more frequently were more depressed and had higher hostility and interpersonal sensitivity at baseline. Satisfaction with the app was high and qualitative feedback provided insights regarding feature enhancements. This research suggested that A4i is feasible in terms of outcome and process indicators and is a technology that is ready to move on to clinical trial and validation testing. This study contributes to the small but emergent body of work investigating digital health approaches in severe mental illness populations.

## Introduction

Schizophrenia and other psychotic illnesses represent a major healthcare challenge globally, accounting for large healthcare expenditures and challenged outcomes for those affected due to the limitations of available treatments. For example, schizophrenia is responsible for 3.8% of hospital admissions in Canada and accounts for an estimated annual cost of 6.85 billion dollars in healthcare and lost productivity [1]. Associated challenges include high rates of suicide, low quality of life, poor access to non-pharmacological interventions, and pharmacological interventions that have suboptimal impacts on community functioning and significant side effects [2]. Overall, schizophrenia has proven extremely difficult to treat effectively, as is amply evidenced by high relapse and re-admission rates [3]. The most common contributors to relapse in schizophrenia are medication non-adherence, social isolation, and inadequate supports [4–6]–problems driven to a large extent by system of care shortcomings [2] and the many challenges presented by symptoms.

The past decade has seen an exponential increase in the development of mobile health (mHealth) strategies to address chronic physical and mental health conditions, with development in the latter concentrating on anxiety and depression apps and platforms [7]. While there has been criticism that the majority of such products provide no evidence for their feasibility or effectiveness, emerging evidence is nonetheless supporting the effectiveness of well-designed platforms in enhancing treatment adherence and illness self-management in a cost-effective manner [8].

Relative to conditions such as depression and anxiety, mHealth for schizophrenia is in a much earlier stage of development. Most of the schizophrenia apps developed to date have only been available in research contexts and very few are commercially available. The lack of technology development in this area is at odds with seemingly strong potential drivers such as illness burden, population size, and formal service gaps which in other contexts have driven mHealth advances. Such a slow uptake of mHealth in schizophrenia might in part be due to assumptions that attend the illness, stigmatizing or otherwise, that include concerns about cognitive capacity, symptoms such as delusions, and poverty, and their implications for technology use. Such assumptions are increasingly being contrasted with findings that over 80% of individuals with schizophrenia and other psychoses routinely use cellphone technology without difficulty [9], with a majority expressing an interest in mHealth interventions [10]. Specific areas of interest for applications include reminders regarding medications, check-ins with practitioners, reminders about appointments, and psychoeducation [11,12]. Aside from schizophrenia-specific content, nearly half in one recent survey have been found to use native apps on their mobile devices to assist with coping, such as listening to audio files to help block voices [13,14].

With respect to the academic literature, a recent review identified 11 studies of schizophrenia-specific mHealth applications [15], with our identifying 4 papers published since [16–19]. These studies, and the broader literature that includes web-based platforms, suggest that the range of ehealth and mHealth approaches that include functions such as reminders, self-assessments, behavioural prompts, and in some instances cognitive remediation, are feasible with this population and do not result in any noted risks [10,15,19–21]. Feasibility studies form the bulk of this small body of literature. Across reviews only three randomized trials have been identified. One trial of text-based weekly symptom monitoring showed no difference in hospitalization over a 1-year period, though had protocol adherence issues [22]. Another large trial of automated text messaging (generic messaging regarding medication adherence and appointments) did not demonstrate significant effects in re-hospitalization and other metrics [23]. One trial by Schlosser and colleagues [18] is the most pertinent to the present study. In this trial, compared to a no-intervention control condition, participants noted over a 3-month period of use improvement in some social engagement metrics (e.g., effort expended). Also reported were reduced depression and negative cognitions with gains maintained at follow up. No change in psychosis symptoms, quality of life, or functioning were observed. Finally, economic assessments and implications for scaling up out of single study contexts to clinical “real world” contexts are not adequately represented in the literature to date [15].

Across the feasibility studies conducted to date, two are particularly relevant to the present study in that they involve a multi-function mHealth platform. The most researched of the two is the FOCUS app which has been developed by Ben-Zeev and colleagues [21]. FOCUS has features that include prompts/reminders with respect to daily activities, brief self-assessments, and tips with respect to coping strategies. Preliminary investigation of FOCUS indicated no risks associated with its use, sustained use over several months, with less use of self-initiated functions. A version of FOCUS that relies on video-based content over text content has also recently shown a good degree of acceptability among users in a pilot study [16]. FOCUS involves users being provided with smartphones with data packages. Another multi-function mobile approach that is in early stages of investigation is Schlosser and colleagues the PRIME app [18], the trial of which is described above. PRIME incorporates motivational texts, goal setting, and coaching by Masters level clinicians, and likewise has shown promising feasibility findings. PRIME relies partially on smartphones being provided to users in tests done to date.

Given the burden of illness that attends schizophrenia and the indicators that mHealth approaches might have an impact in this area, there is a clear need for considerably more evidence in areas of implementation, outcomes, feasibility, and effects. Described here are the development process and outcomes from a novel mHealth strategy for schizophrenia and other psychoses called App4independence (A4i). While having some commonalities with other technologies developed to date with respect to functions such as reminders, text-based coping tips, and peer-peer networking, A4i has some unique features. These features include an ambient sound tester to allow users to discriminate ambient sound from auditory hallucinations. Other key points of functionality include a design that is supported on a range of mobile phone platforms on the users own phone and functionality that accommodates both data plans and reliance upon wifi. These latter elements are essential for populations within which poverty is very prevalent and service contexts where providing phones with data plans for patients is not feasible. This paper describes the initial feasibility testing of A4i in a psychosis population and, as such, contributes to the emergent body of work in this area. This feasibility study examined A4i use over a one-month period using a pre-post, multiple-method design and was intended to form the foundation for future studies of effectiveness and multi-site validation.

## Materials and methods

### Overview

This study was designed to address questions of feasibility and implementation for A4i. The study employed a pre-post design, examining implementation, clinical outcomes, and satisfaction outcomes associated with the use of A4i by individuals with psychosis over an approximately 1-month period. As prompted by Torous and colleagues in their review of mHealth technologies for schizophrenia [15], we report findings using the World Health Organization mHealth Evidence and Assessment (mERA) checklist [24].

### Participants and recruitment

This study took place in a large Canadian urban centre. Several recruitment methods were employed, including (i) recruitment through a centralized process in an early intervention psychosis service in a large tertiary psychiatric facility, physician referral, recruitment from other research programs outside of the centralized process at the same facility, (ii) poster based recruitment across all services at the same facility, (iii) poster-based recruitment through two family support organizations, (iv) poster-based recruiting through a vocational clubhouse, and (v) prior participant peer-referral. The study was reviewed and approved by an institutional Research Ethics Board and all participants completed written consent. The protocol was registered with clinicaltrials.gov (NCT03649815) at the time of submission–this post hoc registration was completed at the request of the journal with pre-implementation registration not completed due to this being a small study of feasibility with no control condition. The authors confirm that all ongoing and related trials for this technology are registered. Participant inclusion criteria included requirements that they be 18 years or older, have a schizophrenia spectrum illness or other primary psychotic disorder as determined through chart review and structured diagnostic interview, and own and use an Android or iPhone mobile device. Capacity to consent was determined through a series of questions to ensure that participants understood the purpose of and their role in the study, their rights with respect to research, what the risks and rewards of the study were and, if possible, through consultation with a referring party. Participants were recruited between April 18^th^, 2017 with the final assessment completed on May 8, 2018. The initial design was to follow a one-week beta test with 60 participants subsequently using the app for 3–4 weeks with pre-post assessments. Some recruitment challenges and a delay in completing the iPhone version led to the study being completed with a total of 38 participants.

### Data collection and measures

After screening and consent procedures, participants completed measures pre and post A4i use. Participants also completed weekly check-in calls (10–15 minutes) to gather more nuanced qualitative data on day-to-day use. They were paid $30 for the initial assessment, $10 per weekly call, and $25 for the post A4i use assessment. Those with limited data plans were also given $25 to offset any additional data streaming costs incurred through A4i use. All payments were in Canadian currency. The A4i server collected app use, post, and within app self-report metrics. A4i was uninstalled from participant phones upon study completion.

Chart/referrer diagnosis was confirmed with the schizophrenia spectrum module of the Structured Clinical Interview for DSM-5 (SCID-5) [25]. Basic demographics were recorded (age, gender, ethnicity, level of education, age of first illness onset) as were details regarding mobile technology use, with their indicating, in minutes, how long on average they used (i) their mobile device(s) generally, and (ii) proportions of time spent texting, gaming, internet browsing, using the camera, and other purposes. These ratings were added to create a composite score of mobile technology use at baseline. Quantitative outcome metrics included (i) the Brief Symptom Inventory (BSI) [26] to assess psychiatric symptomatology, (ii) the Personal Recovery Outcome Measure (PROM) [27] to assess degree of engagement in the recovery process, and (iii) the Brief Adherence Rating Scale (BARS) [28] to examine implications of A4i for medication use. Additionally, at the post evaluation, the mHealth use and utility scale used by Ben-Zeev and colleagues [29] was employed with minor modification to specify A4i use. *Qualitative* data included the use of field notes to capture information gathered in weekly check-in contacts and, at post assessment, a semi-structured interview was used to capture what was more and less helpful/engaging in participants’ experiences of using A4i. Metrics collected *through the app* included overall time used, time and frequency of using specific functions, frequency of app refresh, a sleep-proxy metric (time that the phone is dormant), and daily wellness check-in responses. Descriptive data analysis included a descriptive profile of participants, their app use and ratings of app utility. Outcome metrics were examined using linear regression models including both an intercept only model that is equivalent to paired sample t-test, and a more comprehensive model controlled for demographic covariates (gender, age) and baseline symptomatology. Residual-based model diagnostics were conducted to check for violation of normality assumptions and to detect highly influential cases and outliers of extreme values. Cohen’s d for repeated measures was used to assess effect size. The Holm-Bonferroni method was applied to correct for multiple comparisons. Qualitative analysis was completed using a content analysis procedure [30] to identify common themes, with the theme structure analyzed by a second investigator and minor differences in interpretation negotiated.

### A4i –development and functionality

#### App development and user feedback

The development of the app was prompted by clinical contacts between the first author and an individual with schizophrenia making an effort to use native apps to assist with self-care in the absence of any psychosis-specific apps. This led to literature and market reviews and the engagement with an eHealth provider to scope out the possibility of developing a mHealth solution in this context. The next step was a focus group series with individuals with psychosis, family supports, psychiatrists and case managers to review and give feedback on paper prototypes. The first beta version was built and then tested by five individuals with psychosis for one week, examining use and utility metrics. This information led to enhancements to the app and the delivery of the version described in this paper. Further user feedback was gathered through the study methods described above. Additionally, in the course of developing initial posts to populate the peer-peer feed, one half day focus group was held with individuals with psychosis to brainstorm posts to enter into A4i.

#### Technology platform

The cross-platform software application for mobile devices was created by the A4i development team and is available to install on any smartphone that supports Android or iOS. It is custom software that is not publicly available. The app consists of two main types of features: real-time features such as the newsfeed and offline features such as the toolkit and voice detector. A web API based on the Model-View-Controller (MVC) framework featuring Auth2 security level is used for secure and fast communications between real-time app features and the database. Further, Cordova plugins are used to access native features of the smartphone such as the microphone, accelerometer, and camera.

#### Intervention delivery

For the purposes of this feasibility test, A4i was uploaded onto participant phones by research personnel following the pre-assessment. A web-based portal facilitated A4i setup, including questions in key symptom, need, and goal domains to facilitate the customization of content delivery. The delivery of A4i features in-app and push notifications for daily wellness and goal attainment check-ins that inform content delivery and highlight mental health trajectories. Daily check-ins are delivered twice a day at 9:00 AM and 9:00 PM and survey the user on her/his mental health and/or goal progress respectively. Medication, appointment and event reminders are custom set by the user during onboarding and can be added throughout app use. Medication reminders can specify the exact time or time range as well as day of delivery of the reminder whereas event and appointment reminders can be set to go off at 15 minutes, 2 hours and/or 1 day in advance. If the user does not reply to the push notification from the app within one hour, they will receive an SMS text reminder prompting them to open the app to respond. For medication reminders, users are asked to respond with a confirmation of having taken or not taken the medication. For appointment and event reminders, users can confirm or snooze the reminder by 15, 30 or 60 minutes.

#### Intervention content

A4i functionality includes (see also https://youtu.be/GnzxIuOpPJg): (i) Addressing social isolation through personalized prompts, scheduling of activities, and connections to a range of resources relevant to social engagement. (ii) Fostering engagement in the recovery process through evidence-informed content that makes suggestions and provides resources relevant to coping with psychosis symptoms, negative symptoms of schizophrenia, cognitive challenges, motivation and anxiety as relevant to the individual. The content concentrations are determined by the above-mentioned algorithm. (iii) A peer-peer engagement platform that facilitates strategy/tip-sharing between A4i users (anonymous and moderated). (iv) Daily wellness and goal attainment check-ins to inform content delivery and highlight mental health trajectories. (v) Passively collected data on phone use as a proxy for sleep and activity levels.

#### Interoperability–health information systems context

The next step for the A4i mHealth intervention is to integrate it into existing health information systems (HIS) in the clinical environment for providers to be able to remotely monitor their patients using the app. The integration can be made possible using the Substitutable Medical Applications and Reusable Technologies (SMART) App Launch Framework which uses the Fast Healthcare Interoperability Resources (FHIR) standard developed by Health Level-7 (HL7), an organization that sets standards used for the transfer of clinical and administrative data across the healthcare system. SMART on FHIR has the capability to run applications on electronic health records (EHRs), solving the interoperability problem and allowing clinicians to use the app within their EHR environment. At present, for A4i, this function has yet to be tested.

### A4i –context, cost, security, and other considerations

#### Population level infrastructure

In the large Canadian urban centre where this test was completed, public and private access to electricity is nearly universal and reliable and coverage by the 4 major networks is 100% [31]. It is of note that 13.1% of participants did not have data plans, requiring free WIFI to use all A4i features. In this urban centre there are many sources of free WIFI spread across the city (e.g., coffee shops, libraries, hospitals, community centres).

#### Access of individual participants

The most salient access considerations are the high rates of unemployment [1] and associated poverty that are present among schizophrenia populations. While smartphone access appears to be similar to rates observed elsewhere [9], many do not have access to data packages. This requirement was addressed by A4i to accommodate those who use free WIFI sites by providing offline capability. A4i is resilient to different network situations and is able to store data directly on the mobile device in the event of no network connectivity. Once the app regains network connectivity, data is sent to the database server. The broader consideration of marginalization would include awareness of mHealth options and support in using them. This consideration would be addressed through its provision through care provider services and networks (currently under development for A4i).

#### Cost assessment

While a formal economic analysis has not been completed, the costs of A4i provision for technology delivery and support is estimated at $22,800 per site per year based on a monthly application hosting cost of $800 and a monthly application support, security, and monitoring cost of $1,100. Further, there is a per-patient per-year licensing fee cost of +/-$100. Both estimates are based on the pilot infrastructure and model for this feasibility study. Full implementation including integration, reporting functionality, and predictive modelling is not included in this cost estimate. Not enough validation information has been collected to date to meaningfully assess costs to care providers.

#### Adoption inputs and contextual adaptability

As described above for the purposes of this study. It is anticipated that adoption at scale would be largely mediated through care provider networks that purchase licenses to the technology. The technology has not yet been translated to languages other than English nor has the content been culturally translated aside from attending to diversity in the content streamed to end users.

#### Data security and compliance with national guidelines and regulatory statutes

For the purposes of this research data was stored in accordance with Canadian Tri-Council Policies on the ethical conduct of research. A4i data security was in accordance with both Ontario provincial legislation and Personal Information Protection and Electronic Documents Act (PIPEDA) and the Health Insurance Portability and Accountability Act (HIPAA). All technology is redundant and located at two duplicate networks; switching cores are located in Toronto, Ontario with a hot backup site in Maple, Ontario. Primarily, a .net stack is used, executing Linux implementations for specific purposes. The MEMOTEXT core ‘Sentinel’ engine, which consumes and acts upon A4i data, runs on SQL server with .net hooks for both internal and external data processes. Further, MEMOTEXT follows the Extract, Transform, and Load (ETL) process. Depending on how client data is provided, it can be extracted in different ways. To access an organization’s assets, web APIs and secure FTP where files are encrypted with PGP keys are used. The data is loaded into an MS SQL or MySQL database where further processing occurs; table level encryption is utilized and the transmission of data is protected by TLS 1.2 where required. Only internal personnel may access data from secured locations with security being facilitated by two level password access—VPN and Domain–with no synchronization of password between layers. MS Best Practices are followed with respect to password complexing and having a 90-day expiration cycle.

## Results

### Participants

**Fig 1:**
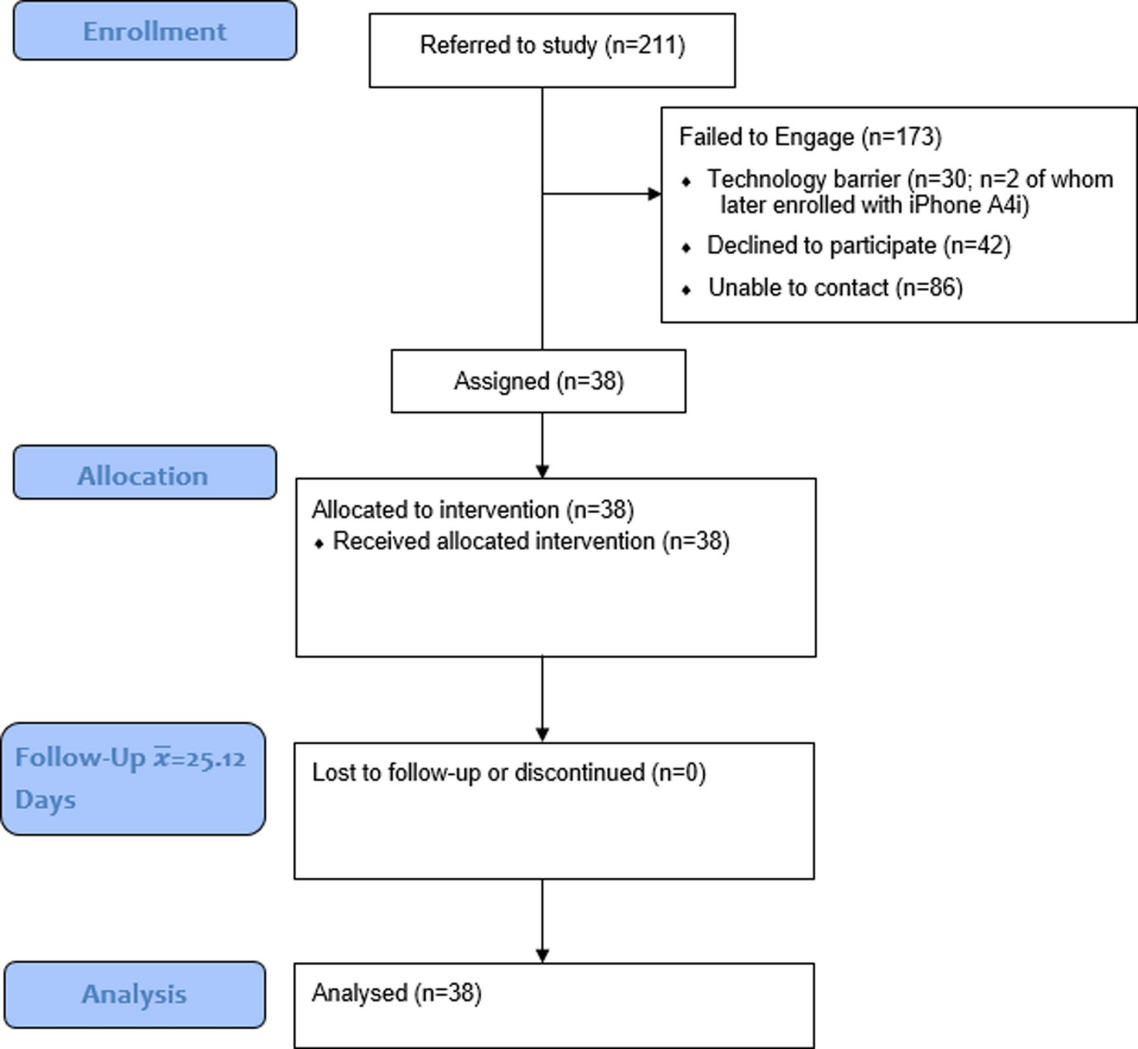
Consort diagram.

A total of 211 individuals were referred to the study having met screening criteria through a centralized research intake process. Of these 211 referrals, 173 did not participate in the study. The primary reason for referred individuals not participating was an inability to contact them to schedule the consent and assessment despite multiple emails and calls (n = 86). As most referrals came from a high acuity psychosis early intervention service (both inpatient and outpatient-sourced referrals), it is likely this reflects the broader challenge with engaging this specific population. Of those contacted successfully, some (n = 30) did not own the appropriate technology (e.g., Blackberry device, iPhone before the iPhone version was ready 8 months after the Android version– 7 of whom were waitlisted and of which two were enrolled when it was available; two people could not be contacted, one had changed phones, and two were unsure/too busy to participate and declined.). Some declined participating after learning more details about the project (n = 42), with n = 15 miscellaneous reasons for not participating (see Consort diagram, Fig 1). Due to a lack of consent we are unable to report on the demographics of those who couldn’t be engaged or chose not to participate. The total number of participants who used A4i was 38—of whom 27 (71%) identified as male, 10 (26.3%) female, and 1 (2.6%) transgender (Table 1). There was no attrition. Sixteen identified as of White European origin, 9 as Black Canadian of African or African Caribbean origin, 2 South Asian, 1 Indigenous, and others mixed. Mean participant age was 31.4 (range 19–61) with 63% having a primary schizophrenia diagnosis (n = 24), 23.7% schizoaffective (n = 9), and the remaining having psychosis NOS or psychosis comorbid with bipolar disorder (n = 1) and autism with prominent psychosis symptomatology (n = 1). Most lived alone in a private dwelling (34%), followed by private dwellings with parents (24%) or with roommates (24%). With respect to education, most had completed some college or university courses (73.7%), followed by high school graduates (15.8%), with scatter across other categories above and below. Android devices were used by 76% of participants with 24% using iPhones. Considering mobile technology use at baseline, n = 30 reported hourly use and n = 8 daily, with most using core functions 1–5 times/day (texting, n = 20; email = 21; social media = 16). It is of note that BARS data was not collected for two participants due to their not being prescribed psychiatric medication at the time of the study. Note that quantitative and qualitative data is available via S1 and S2 Tables respectively.

**Table 1:** Demographic profile.

			(N = 38)			
			N	%		
**Categorical Variables**						
	Gender					
		Transgender Male	1 27	2.6 71.1		
		Female	10	26.3		
	Race and Ethnicity					
		White	16	42.1		
		Black of African or Caribbean Origin	9	23.7		
		Mixed	9	23.7		
		Other	4	10.5		
	Level of education					
		Junior High/Middle School	4	10.5		
		High School	6	15.8		
		Tertiary Education	28	73.7		
	Diagnosis					
		Schizophrenia	24	63.2		
		Schizoaffective	9	23.7		
		Psychosis NOS	3	7.9		
		Bipolar	1	2.6		
		Autism (with psychosis symptoms)	1	2.6		
	Employment					
		Full Time	2	5.3		
		Part Time	6	15.8		
		Casual	4	10.5		
		Student	11	28.9		
		Unemployed	9	23.7		
		Not In Labour Force	6	15.8		
**Continuous Variables**			Mean (SD)		**Range (min-max)**	**N**
	Age		31.42 (8.60)		19–61	38
	Age at 1^st^ Hospitalization		24.16 (7.70)		14–52	n = 37, one participant never hospitalized

### A4i use

In the course of this feasibility test A4i was used for a mean of 25.12 days (SD = 6.32; range: 16–46 days; Table 2). The outliers accounting for this range were due to some of the offboarding scheduling challenges that attend individuals with severe mental illnesses (e.g., conflicting appointments, challenges with arranging travel, difficulty booking appointments). Controlling for days of use, the mean classic retention rate was 52.50% (SD = 22.13). The classic retention rate is defined as the percent of total app users that come back to the app on a specific day–this was calculated daily and averaged for the study period. The total number of active (response to or initiation of any activity) interactions with the app ranged from 6 to 654 times in the exposure period. The mean number of interactions per day was 4.21 (SD = 5.19; Median (M) = 2.20). Rolling retention/churn rates (rates of users coming back to the app) for 7 days was 100% and for 20 days (excluding n = 2 who completed app use prior to 20 days) was 94% with 6% churn (2 users did not return to the app on or after day 20). With respect to the core function of the app, using the feed to like, post, or save content, in the first 7 days 37/38 users used the feed a mean of 4.49 (SD = 12.25; M = 1.86,) times per day and within the first 20 days, 36/38 (n = 2 excluded) used the feed a mean of 2.66 (SD = 4.83; M = 1.48,) times per day. Skewness in the app usage data due to several users who were heavily using the app led to high standard deviations; the median is thus a more appropriate summary statistic to be examined in the above app use results.

**Table 2:** A4i use.

**Measure**	**Statistic**	**Error**	**N users**	**Period of use**
Mean classic retention rate	52.50%	SD = 22.13	38	Controlled for days of use, 16–28 days
Number of interactions per day	Mean = 4.21, Median = 2.20	SD = 5.19	38	Duration of app use
7 day rolling retention/churn rate	100% returning, 0% churn	NA	38	First 7 days of app use
20 day rolling retention/churn rate	94% returning, 6% churn	NA	36	First 20 days of app use (2 completed prior)
Using the feed–first 7 days (times per day)	Mean = 4.49 Median = 1.86	SD = 12.25	37	First 7 days of app use
Using the feed- first 20 days (times per day)	Mean = 2.66 Median = 1.48	SD = 4.83	36	First 20 days of app use
**The total number of active (response to or initiation of any activity) interactions with the app**	**Range: 6–645 times**	**NA**	**38**	**Duration of app use**

### Outcome findings

Considering the outcome analysis without controlling for covariates (Table 3 and S1 Fig), significant improvements were observed for Psychoticism (*p* = .04, d = 0.22); Depression (*p* = .001, d = 0.42), Phobic Anxiety (*p* = .02, d = 0.23); Obsessive Compulsive (*p* = .01, d = 0.38); Paranoid Ideation (*p* = .009, d = 0.29); and Interpersonal Sensitivity (*p* = .04, d = 0.18). However, applying the Holm-Bonferroni correction for multiple comparisons yielding stepped significant levels starting from .005, only depression remains as having a significant subscale reduction. One highly influential case was removed from the BARS as a result of model diagnostics. With that outlier removed, there was a significant pre-post improvement in BARS adherence scores (*p =* .03; d = 0.21) however this improvement needs to be interpreted with caution due to the outlier issue, the ceiling effect on this measure, and its’ not remaining significant after controlling for multiple comparisons (S1 Fig). There was a significant improvement in recovery engagement measured by the PROM (*p* = .047, d = 0.19; S1 Fig), however this finding did not remain significant after correcting for comparisons. In model 2 we controlled for demographic covariates (gender, age) and baseline symptomatology (Table 4). This model, after correcting for multiple comparisons, resulted in findings of significant declines in the depression subscale as was the case in model 1 (p < .001), in addition to Obsessive Compulsive (p = .004) and Paranoid Ideation subscales (p < .001). The possibility of including, as covariates, hospitalization data in a third model (number of hospitalizations, years since first hospitalization) was probed. It is not included here, however, as this analysis did not add significance and adding these additional covariates, given the sample size, brings a risk of reporting errors.

Looking at the measure of satisfaction with app functions, measured on a scale of disagree, neutral, and agree, most responded agree to most items (mean = 68.32%; range 37%-84%). The strongest endorsements occurred in items tapping ease and comfort of use. The weakest endorsements fell in items speaking to “need” to use A4i and wanting to use A4i “often”. Splitting the participants into low and high daily user groups, based on position above or below the median, identified n = 18 in each group. Using independent samples t-test to then compare low and high users on outcomes, no difference was observed for the PROM, BARS, total BSI nor BSI subscales. Comparing baseline scores as a function of A4i use, while PROM, BARS and total BSI scores were not significantly different, there were differences in BSI subscales. Specifically, more frequent A4i users were at baseline more depressed ($\overset{-}{X}$ = 8.33 (SD = 5.81) vs 4.80 (SD = 4.95); (t = 2.02, df = 36, p = .05), had higher hostility ($\overset{-}{X}$ = 3.33 (SD = 2.59) vs 1.60 (SD = 1.96); (t = 2.34, df = 36, p = .03), and reported more interpersonal sensitivity ($\overset{-}{\overset{-}{X}}$ = 5.94 (SD = 4.16) vs 3.10 (SD = 3.14); (t = 2.38, df = 36, p = .02). Considering associations with the composite score of baseline (non-A4i) mobile technology use ($\overset{-}{\overset{-}{X}}$ = 20.40 (SD = 6.61), no significant correlations were observed with change in PROM, BARS, or mean BSI scores nor was there a significant difference in baseline mobile technology use as a function of membership in the high or low A4i user groups (t = 0.94, df = 36, p = 0.36).

**Table 3:** Pre-post ($\overset{-}{\overset{-}{X}}=25.12\;days)$ change (Paired sample t-tests).

Outcome Measure	Baseline Mean (SD)	Post Mean (SD)	95% CI	t	p-value	Within group Effect Size (*Cohen's d*)
	*n = 38*	*n = 38*				
Brief Symptom Inventory						
Total	46.21 (32.86)	41.66 (33.80)	[-0.33, 9.43]	1.89	0.067	0.14
Psychoticism	5.34 (4.63)	4.34 (4.23)	[0.06, 1.94]	2.16	0.038	0.22
Somatization	4.95 (4.40)	4.29 (5.40)	[-0.54, 1.85]	1.11	0.273	0.13
Depression	6.47 (5.59)	4.29 (4.57)	[0.97, 3.39]	3.66	0.001	0.42
Hostility	2.42 (2.41)	1.97 (2.58)	[-0.1, 0.99]	1.67	0.104	0.18
Phobic Anxiety	3.90 (4.11)	2.97 (3.75)	[0.13, 1.72]	2.35	0.024	0.23
OCD	8.21 (5.06)	6.37 (4.70)	[0.47, 3.21]	2.72	0.010	0.38
Anxiety	4.92 (5.50)	4.63 (4.81)	[-0.84, 1.42]	0.52	0.606	0.06
Paranoid Ideation	4.97 (4.54)	3.71 (3.49)	[0.34, 2.19]	2.77	0.009	0.29
Interpersonal Sensitivity	4.45 (3.90)	3.74 (3.94)	[0.02, 1.40]	2.08	0.044	0.18
Brief Adherence Rating Scale	98.27 (3.10)	98.94 (2.35)	[-1.2, -0.06]	-2.23	0.032	0.21
Personal Recovery Outcome Measure	7.13 (1.66)	7.45 (1.63)	[-0.63, 0.00]	-2.05	0.047	0.19

Cohen’s d for repeated measures used: $d=\frac{{\overset{-}{y}}_{2}-{\overset{-}{y}}_{1}}{s_{z}}$, where $s_{z}=\sqrt{\frac{s_{1}^{2}+s_{1}^{2}-2rs_{1}s_{2}}{2(1-r)}},\;s_{1},\;s_{2}$ and *r* are standard deviations of the pre and post measures and their correlation.

**Table 4:** Model 2 output (Controlled for gender, age and baseline symptomatology).

Outcome Measure	t	p-value
Brief Symptom Inventory		
Total	1.87	0.071
Psychoticism	2.31	0.027
Somatization	1.14	0.262
Depression	4.36	<0.001
Hostility	1.72	0.095
Phobic Anxiety	2.52	0.016
OCD	3.04	0.004
Anxiety	0.53	0.598
Paranoid Ideation	3.45	0.002
Interpersonal Sensitivity	2.07	0.046
Brief Adherence Rating Scale	-2.93	0.006
Personal Recovery Outcome Measure	-2.18	0.036

### Qualitative findings

Participants, commenting on what they believed was the primary objective of A4i, emphasized both scheduling and reminder functions “easily reminding me about the next time I need to take my meds”, and strategies to manage symptoms “[it helps me] redefine my daily thoughts…for people to feel mentally healthy”, “helps you focus on something when your thoughts are racing”, “mainly its used to dissuade feelings of hopelessness or the negative symptoms of schizophrenia.” Others commented on peer-peer connections, being provided about information in the community, and noted that the “sound detector is useful for figuring out what’s real.” Feedback on the design was mixed. While some thought that no improvements were needed, some commented that they would have rather a different color scheme or one that is customizable, that the “layout could function more smoothly”, and that the design felt “old school.” Favorite features were predominantly scheduling and reminders, being able to record notes for their doctors, “the feed” in which they could learn about and share strategies, and the ambient sound detector to determine if they were having a hallucination. Dispersion across similar areas that emerged in comments on features that weren’t necessary spoke to the need for customization. Some commented that the ambient sound detector wasn’t necessary since they didn’t have hallucinations some noted that scheduling and notes to doctor functions weren’t relevant for them. One individual commented that they wanted a more robust means to provide feedback about A4i other than a thumbs up/down about how A4i is working for them. Eleven participants did not find any of the features redundant or otherwise unnecessary. Only one participant noted a negative response aside from broadly disliking a feature or finding a feature not useful. This individual noted anxiety regarding text messages which made this person feel that someone was “monitoring” her. However, this same person also made positive remarks and noted that if A4i were to become available they would “definitely” use it. Aside from this finding no unintended or adverse events were observed related to A4i use. With respect to inquiry about what participants would change about A4i, approximately one half of the comments highlighted desired functions (“add noises to reminder banners”), one third noted desired improvements in the colour scheme and layout, and one third emphasized enhancements to the peer-peer feed (e.g., ability to “comment on the feed”, “separate feed into stories, resources, and inspiration”, “improve community outreach and interactivity- get more people on it at the same time”).

## Conclusions

This paper describes the feasibility and implementation of a novel mHealth approach for individuals with schizophrenia spectrum illnesses. Work in this area is important given the paucity of research and technology development for this spectrum of illnesses that represent an extremely high healthcare burden [1] relative to the large body of work in areas such as anxiety and depression [7]. The intervention tested here, A4i, employs a multi-feature platform tailored to individual symptoms through an algorithm, operates on the individual’s own Android or iPhone with or without data. This offers some unique conditions that map on well to the nature of the illness and other key contextual considerations related to individual contexts and the largely underfunded service sectors that are available for people with schizophrenia globally.

With respect to feasibility data points, this study demonstrated retention findings that are similar to other comparable interventions such as FOCUS [29]. Comparison with PRIME is more difficult as classic retention was not reported though average rates of function use suggest similar findings [18,23]. It is of note that the rolling retention rate of A4i is substantially better than what is typically observed in the broader mHealth space and, more specifically, with regard to mHealth apps that are prescribed by a provider [32,33]. However, this observation needs to be considered with caution as it may be influenced by app use within the structure of a research study and, in particular, may have been influenced by the weekly check-in contacts. Also similar to previous work (FOCUS and PRIME), we observed some modest symptom reductions with small-moderate effects with decreased depression being the most robust finding. These observations of change were more pronounced after controlling for baseline symptoms, gender and age, though number of hospitalizations and time since first hospitalization likely have much less influence when controlled for. Changes in medication adherence and personal recovery ratings, while positive, did not remain significant after controlling for multiple comparisons. Examining engagement in the recovery process and other positive characteristics such as resilience as was done in this study is likely important in a field that to date has concentrated primarily on symptom reduction. The closest comparator in this area is PRIME for which cognitive variables were examined that might have some overlap with the recovery construct [18]. Finally, the observation that those who most frequently engage the app functions reported higher depression, hostility, and interpersonal sensitivity at baseline has implications for future work that might address function tailoring based on baseline assessment. This observation could also have implications for risk prediction based upon app use analytics.

There are several points of note with respect to assessing the potential for scale and overall viability of the approaches being discussed here–particularly given the under-resourced service contexts in which these technologies would be deployed. First, the outcomes described above were observed for A4i using individuals’ own devices with or without data whereas FOCUS and PRIME involved providing some or all users with smartphones with a data plan. Second, PRIME involves 1–1 coaching by Masters level clinicians, which is a highly resource-intensive feature. However, more information about user engagement with A4i would be helpful. It is unclear about the degree to which those who could not be reached to participate was due to challenges other than an interest in A4i and, for both that group and those who explicitly declined, how much of this decision making was influenced by the app versus the app *and* participating in a research study. These caveats aside, the finding that 33.6% of those successfully contacted declined to participate and upload A4i is consistent with participation rates in other studies of schizophrenia apps [21] and is consistent with mobile health engagement data from non-schizophrenia, mental health populations [34]. One point of particular salience in this regard is the relatively large proportion of male participants–with numbers reflective of neither psychosis populations nor the clinical services at the study site. More data is needed to determine if gender is a factor in engagement with A4i or an artifact of other considerations. Lastly, it may be the case that A4i is of more interest to younger, early illness-course populations given the mean age of 31.4 and being within approximately 6 years of first hospitalization on average. This observation requires more information, however, as it may be an artifact of the recruitment site and methods.

Looking at user feedback, quantitatively we observed similar outcomes to other applications with the majority of users reporting satisfaction with the technology. This was particularly evident regarding ease of use. Users felt less strongly about how much they “needed” the app, perhaps speaking to future work that should examine motivating elements as the technology is brought to scale and validated. Emphases in qualitative feedback suggested the benefit of a multi-function approach give the diversity of illness presentations and needs. Most were satisfied, but it was also highlighted that the look and feel of A4i could be improved. This comment reflects less upon shortcomings in the intended design but more on the highly competitive and challenging environment for mHealth development [35]. It is difficult to obtain beta technology development funding at a level that can compete with the funding that goes into app design for functions that people routinely use and are used to–leaving A4i looking “old school” as one participant noted.

This study had several limitations. As noted, no control condition or conditions limits any comment on effectiveness and limited the ability to control for possible confounders such as baseline symptomatology. As well, while the sample size was adequate for this initial test of feasibility, a larger sample would allow for comment on user sub-populations and speaking to how that affects app use and outcomes. Another limitation is the time period of the test which might speak to a lack of signal in the area of medication adherence, though the test was likely of sufficient length to detect and measure major risks of attrition or retention failure given that both non-prescribed and prescribed mHealth apps typically evaluate retention rates using the standard first 30-day window [32]. This point aside, future work will need to conduct tests of longer time periods as others have done [17,18, 29] to better assess the degree to which the gains observed in this study are sustained. Additionally, this study cannot speak to how well A4i would perform outside of the research context and the context in which it was developed nor could this design for the possible confounds of the treatments participants were engaged in alongside A4i.

Next steps, which reflect both where the study of A4i needs to proceed as well as the modest field to date, include 4 elements–all of which are necessary for this technology to be brought to scale. First, a better-powered randomized trial needs to be completed to address effectiveness and more fulsomely explore the provider portal aspect of A4i. Second, a validation study is needed to examine how effectively A4i can be implemented in other sites–sites that are better reflective of “real world” considerations. Third, methods through which A4i can connect to electronic medical records need to be determined. Fourth, a clear commercialization strategy and economic analysis are needed to build the business case for this technology. A demonstrated return on investment is needed to provide A4i with the funding that is necessary to bring it to scale. These considerations, several of which extend beyond the research domain specifically, are all essential to the success of A4i in terms of demonstrated effects, feasibility, and viability. The latter point is particularly salient for schizophrenia wherein it can be anticipated that the individuals themselves will not be the app purchaser in most circumstances. The rigorous development process may perhaps, along with some stigmatizing assumptions about people with the illness and their ability to use technology, partially explain the relatively early stage of development of mHealth for psychoses.

## Supporting information

S1 Table Quantitative outcome raw data. (XLSX)

S2 Table Qualitative data. (XLSX)

S1 Fig Boxplots. (DOCX)

S1 File Trend checklist. (DOC)

S2 File Study protocol. (DOCX)
