# Supplementary material for: Feasibility and outcomes of a multi-function mobile health approach for the schizophrenia spectrum: App4Independence (A4i)
Source: PLoS One. 2019 Jul 15;14(7):e0219491. doi: 10.1371/journal.pone.0219491 (PMC6629069; doi:10.1371/journal.pone.0219491)
Supplement: S1 Fig — (DOCX) [file pone.0219491.s003.docx]

**Figure 1 BSI Outcomes**

**
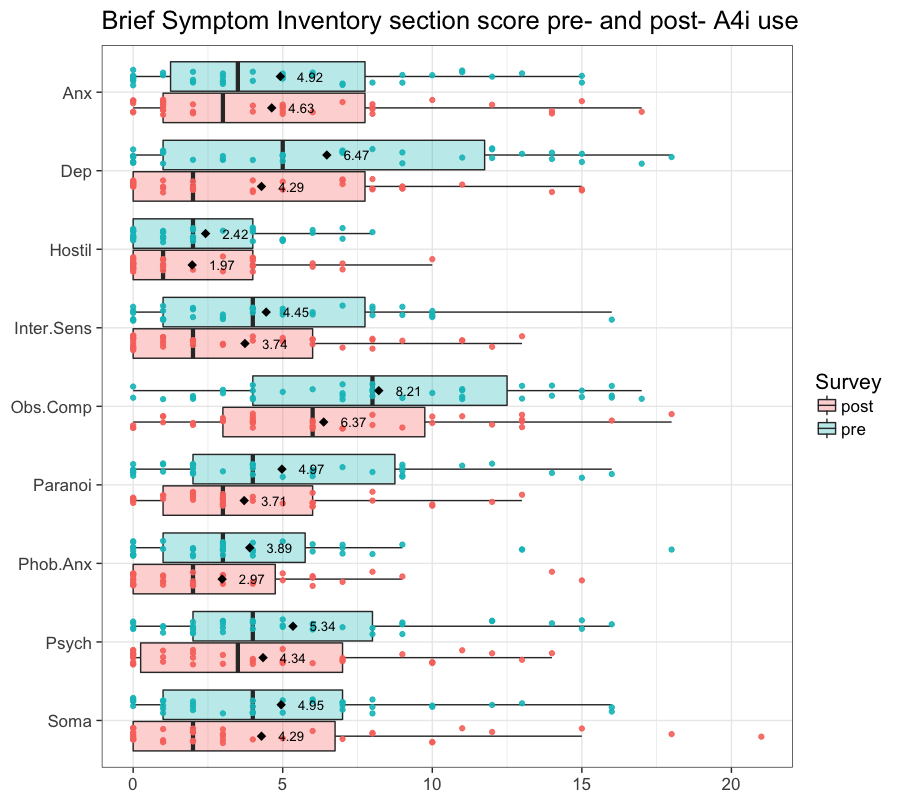
**

**Figure 1. BSI score box plots, per section score**

**Pre- and post-A4i use measures from the Brief Symptom Inventory scale, including psychoticism, somatization, depression, hostility, phobic anxiety, obsessive compulsion, anxiety, paranoia, and interpersonal sensitivity are plotted by score for the 1^st^ and 3^rd^ quartiles, in addition to the median. Whiskers represent the minimum and maximum values, and outliers are represented by circles beyond the end of the whisker. Median scores are indicated by the black vertical line with black diamonds indicating means with values provided. N=38.**

**Figure 2 – BARS Adherence Outcome**

**
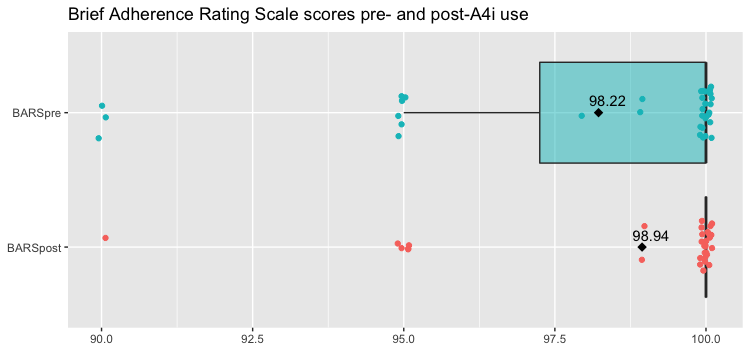
**

**Figure 2. BARS scores pre- and post-A4i use. N = 36.**

**Figure 3 : Personal Recovery Measure Outcome**

**
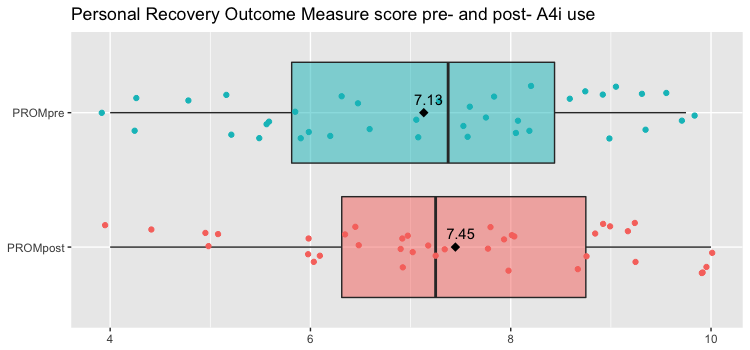
**

**Figure 3. Personal Recovery Outcome Measure pre- and post-A4i use. N=38.**
