# Supplementary material for: Feasibility and outcomes of a multi-function mobile health approach for the schizophrenia spectrum: App4Independence (A4i)
Source: PLoS One. 2019 Jul 15;14(7):e0219491. doi: 10.1371/journal.pone.0219491 (PMC6629069; doi:10.1371/journal.pone.0219491)
Supplement: S2 File — (DOCX) [file pone.0219491.s005.docx]

**STUDY PROTOCOL**

**(Original – 8 September 2016)**

**(Version 2 – 4 April 2017)**

**(Version 3 – 28 April 2017)**

**(Version 4 – June 20 2017)**

**(Version 5 – October 20 2017)**

**Study Title: Testing the Feasibility and Outcomes of a Digital Health Support for Individuals with Schizophrenia Spectrum Mental Illnesses**

**Principal Investigator: Sean Kidd, Ph.D.**

**Co-Investigators: Kwame McKenzie, MD, Aristotle Voineskos, MD, Ph.D., Yukari Seko, Ph.D.**

**Introduction:**

**Overview**

This protocol describes an attempt to capture the development phase of a mobile support for individuals with schizophrenia. The intent is to describe and account for a rigorous development process that will result in the creation of a beta version that would be tested in a randomized trial for effectiveness - to be addressed in a subsequent protocol

**Background**

*General Issues*

The most common contributors to relapse in schizophrenia and associated mental illnesses are medication non-adherence, social isolation, and inadequate supports^1-4^. Driven to a large extent by system of care shortcomings^5^ and the many challenges presented by symptoms, the impacts of these problems are profound from individual to system levels. Schizophrenia is responsible for 3.8% of hospital admissions in Canada and accounts for an estimated annual cost of 6.85 billion dollars annually in healthcare costs and lost productivity^6^. Associated challenges include high rates of completed suicide, low quality of life, and care provision issues including poor access to many evidence based non-pharmacological interventions and pharmacological interventions that are having only modest impacts on community functioning and quality of life in the current system of care^5^.

This is a global issue, and to date technology has not been substantively leveraged in generating solutions - despite evidence of substantial uptake of relevant technologies by relevant populations. For example, it has been found that approximately 70% of individuals with schizophrenia routinely use cellphone technology without difficulty^7^. To date, there are no products on the market that address the constellation of issues outline above. It is an area where a nuanced approach is needed as this illness is highly diverse in presentation, attended by a number of social determinants of health that greatly affect outcomes and, quite commonly, ambivalence with respect to service provider and caregiver engagement. This scenario as it exists for schizophrenia stands in sharp contrast with the many thousands of applications developed for other mental health issues^8^.

*Cellphone Based Technologies and Schizophrenia*

While there are no marketed digital supports to address medication adherence and social isolation (among other, related concerns) for schizophrenia, a number of investigators have conducted pilot studies of the feasibility of such approaches. First, surveys of relevant populations have suggested that a majority would be interested in mobile interventions (72%)^9^, and other work has indicated that domains considered feasible for applications include reminders regarding medications, check ins with practitioners, reminders about appointments, and psychoeducation^10,11^.

There is a small, emergent literature that is examining the feasibility and outcomes of mobile applications that address schizophrenia. To date these applications have not entered the open market. Broadly, targeted mobile and online applications in areas such as cognitive remediation (brain training games) have been found feasible and do not result in any noted risks in their use^12^. More directly relevant to this protocol is the work of Ben-Zeev and colleagues^9,13^. This mobile phone application has features that include prompts/reminders with respect to daily activities, brief self-assessments, and tips with respect to coping strategies. Preliminary investigation of this application indicated no risks associated with its use, and ready uptake by individuals which schizophrenia. A study of outcome among 33 individuals with schizophrenia spectrum illnesses was somewhat less promising with no indication in symptom change in time of use nor in beliefs about medications and no association between amount of use and symptoms assessed via the PANSS. This application is currently being assessed as integrated into a large early intervention trial in the United States under Dr. John Kane^14^.

*Local Work in this Area to Date*

At CAMH for the past 5 years we have been working in close collaboration with Dr. Dawn Velligan's group at the University of Texas at San Antonio on the study and development of environmental supports/compensatory interventions to offset the cognitive impacts of schizophrenia^15^. This work has recently included an emphasis on a younger, early intervention population (currently an RCT of Cognitive Adaptation Training versus Action Based Cognitive Remediation underway). These experiences have led to an appreciation for how compensatory strategies might leverage technologies both older (e.g., multi-alarm, voice recording clocks) and newer (e.g., setting up reminders on cellphones). Initially our focus was upon medication delivery devices, though patent, marketing landscapes and ambiguous evidence for same suggested that mobile technologies were more promising.

This led to a collaboration with MemoText (www.memotext.com) and preliminary design efforts to develop a digital heath approach for schizophrenia - henceforth referred to as A4i or App4Independence. Memotext is a company that, via our collaboration with its founder Amos Adler, has a large amount of experience in the generation of evidence based digital health approaches for chronic illnesses. While to date mental health has not been a focus, its platform is conducive to translation to a schizophrenia application and the partnership with CAMH facilitates the relevant expertise.

Initial mapping of key domains relevant to an app in this area has been conducted based upon the experience of the collaborators and an understanding of the relevant practice literatures. This initial draft, which might be considered a 'paper prototype', involves the following:

The platform will:

- Help prevent social isolation through personalized prompts, scheduling of activities, and connections to a range of resources relevant to social engagement
- Enhance hopeful and informed engagement in the recovery process through functions that foster resilience and draw on evidence based strategies to enhance wellness (e.g., personalized affirmations; tip sheets; relaxation exercises)
- Facilitate automated support and link to caregivers
- Encourage and check-in on daily essential activities for patients - addressing memory, attention, and initiation challenges that often occur as a part of this illness
- Provide basic health/safety functionality and track level of wellness

The mobile solution will use a combination of intake and ongoing assessments, accelerometer, geolocating and mobile based sensors to assist in mapping activity levels in accordance with participant’s goals and could begin to inform tools that might address relapse risk detection and prevention.

Specifically the app will be made up of 4 functional areas:

1. A needs assessment in order to determine care/interaction pathway, crisis planning and routine builders, accompanied by ongoing assessment to keep components relevant and engaging and to facilitate treatment planning and support by providers.
2. Daily interactions and check-in functionality for self-determination of messaging, reward messaging, social interaction content.
3. Algorithm to determine content based on ambient and interaction data content triggers and risk flagging built on evidence—based guidelines.
4. Self-Management portal and caregiver dashboard.

The solution will be simple to set up and reflexively and actively engage users in modifications to what is offered. While readily set up and engaged in by some individuals with schizophrenia, the design anticipates that for many a caregiver will assist with set up and will be involved in monitoring and prompting use. Figure 1, below, provides a further description.

Figure 1 - Draft Functional Plan


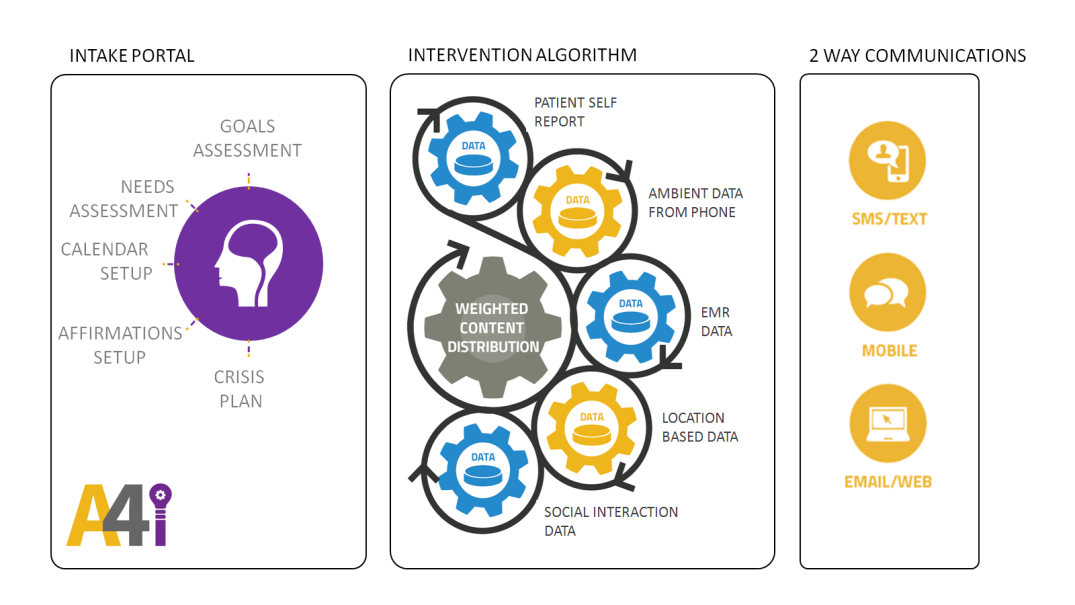


**Research Objective**

The work done to date is conceptual based upon, effectively, thorough and informed guesses as to what would work with this population. It is our intent to engage in a rigorous process of review and development with active feedback from prospective uses of the technology and pilot testing. This will then be followed by a randomized trial of the beta version that will be generated through the process outlined in this protocol (will be the subject of a subsequent protocol and not addressed here). **As such, the objective of this research is to capture and record the process of development of a functional, beta version of this technology.** Our group has been fortunate enough to secure a grant for seed funding from the CAMH Foundation which will facilitate this effort over the coming year.

In coming months Memotext will use its current platform (as mentioned has generated evidence in treatment adherence in other areas of medicine^16^) to generate a working prototype with basic functions in place in the areas outlined above. This initial version of A4i would undergo three stages of formal testing and iteration development (note that the project team will be informally piloting elements of A4i in advance to troubleshoot problems before formal study).

**Stage 1**

In the first stage of testing we would plan to recruit 6 individuals with a schizophrenia spectrum diagnosis to use A4i for a 1 week period. A sample size of 6 will be adequate for this initial, in depth examination of use and utility. With respect to **recruitment**, we will use our existing CAMH case manager network to reach out (Appendix 1) to prospective participants and also would recruit using a flyer (Appendix 2) distributed within CAMH and through organizations such as the Schizophrenia Society of Ontario, specifying that we are seeking individuals who:

- Are 18 years of age or older
- Have a schizophrenia spectrum diagnosis (process would represent confirmation of same)
- Own and regularly use a smart phone equipped with an Android operating system with a talk and data plan
- OR
- Are willing to use a loaner Android phone with a talk and data plan for 7 days in total

1. With respect to procedures, in an initial consenting and assessment appointment (60-90 minutes) the following would take place:

1. Written consent obtained and capacity to consent confirmed.
2. Basic demographics recorded (age, gender, ethnicity, level of education, age of first illness onset)
3. Completion of the 53-item Brief Symptom Inventory (BSI^17^) which will be used to help in the triangulation of the data collected as a function of symptom level and change therein over the study period.
4. Completion of the 30-item Personal Recovery Outcome Measure (PROM^18^) to assess degree of engagement in the recovery process and utilized in the same manner as the BSI.
5. Medication adherence will be assessed through the Brief Adherence Rating Scale (BARS^19^).
6. Social functioning and changes therein will be captured using the Social Functioning Scale which is an approximately 15 minute survey about social and community activities (SFS^20^).
7. Participants will then be asked to indicate mobile technology use indicating quantitatively, in minutes, how long on average they used (i) their mobile device (s) generally, and (ii) proportions of time spent texting, gaming, internet browsing, using the camera, and other purposes and, qualitatively, (i) what they found the most useful/enjoyable in each of these areas and what was less useful/enjoyable.
8. If participants use their own phones, they will be notified both verbally and in the consent form that using A4i will consume some of their cellular data package, and will be offered $25 as compensation for a data top-up to mitigate the chance of data overages.

2. Participants, on the first day of the week of study (which may coincide with provision of consent and completion of the above measures), would take 30-60 minutes for an orientation session and to install A4i on their phone, initiate personalized settings and content, and orient to functions. Entrance will continue on a rolling basis.

3. The app will automatically keep a record of use metrics (total time spent, time spent on specific tools).

4. For weekdays during the trial (4 days in total), brief (10-15 minutes) daily phone interviews (Appendix 3) will be conducted with each of the participants probing overall satisfaction with the app, what elements were more or less helpful, what improvements might be made, and what was not being used and why. Responses will be audio-recorded and transcribed. Research study personnel will send a daily text message reminder to participants to confirm time of phone interviews and in-person appointments (Appendix 4). All text messages will be sent from a CAMH approved cell phone.

5. No prompts or interviews will be done over the weekend to allow for some examination of use without prompting.

6. Seven (7) days after app installation/loan of the phone, a follow up meeting will be held (60-90 minutes) in which the BSI, BARS, and PROM will be repeated, and the modified 26-item use and utility scale used by Ben-Zeev and colleagues (2014) will be employed to facilitate comparability - itself derived from 3 validated measures of online application use.^21-24^ This will be followed by a semi-structured interview using the same prompts as item #4 but reflecting on the week of use as a whole. At the end of the meeting A4i will be uninstalled from their phone (they will be provided with a list of evidence based apps from other areas of mental health that address stress, scheduling, and other relevant domains in the event that this experience has prompted an interest in using such tools going forward).

Reimbursement for participation in the above stage will be $25 for each of the pre and post assessment meetings, $25 for the app setup meeting, and $25/day (inclusive of expectation of app use and brief interviews) for a total of $200.

Data analysis of stage 1 information will be descriptive - comprised of descriptive summary statistics for quantitative measures and demographics, a qualitative content analysis to categorize feedback from interviews, and some probing in a mixed-methods manner differences in use and response as a function of ratings on scales and demographics (e.g., lower versus higher PROM scores).

**Stage 2**

Following the completion of Stage 1, A4i will be revised based upon the information gathered in that stage. This is expected to take approximately one month. In Stage 2 there is a shift from a primary objective of obtaining feedback to a primary objective of testing feasibility while continuing to generate feedback that can inform the next iteration.

In this second stage of testing we would plan to recruit 30 individuals with a schizophrenia spectrum diagnosis to use A4i for a 1 month period. Thirty participants will allow sufficient power for quantitative analyses and will be more than adequate to saturate themes with respect to use and utility.

With respect to **recruitment**, we will use our existing CAMH case manager network to reach out (Appendix 5) to prospective participants, we will coordinate with other concurrent CAMH research projects where participation is complete or ongoing (e.g. Slaight, or via follow-on recruiting permitted by consent to contact participants for further studies at CAMH as per protocols #104-2014 (GAPP) and #163-2011 (IMPACT)), and also would recruit using a flyer (Appendix 2) distributed within CAMH and through organizations such as the Schizophrenia Society of Ontario, specifying that we are seeking individuals who:

- Are 18 years of age or older
- Have a schizophrenia spectrum diagnosis
- Own and regularly use a smart phone equipped with an Android operating system and a talk and data plan
- Read and speak conversational English

We may also ask participants who used the app in the first stage of this protocol to return and use it for the 30 day trial period in the second arm.

In this period we also intend to explore processes that attend ‘real world’ implementation.

The first of two strategies for this include case manager setup and engagement in supporting A4i use – this is a key question that is relevant to ‘real world’ implementation. Accordingly, we will engage our case manager network to determine 3-5 case managers willing to approach 1-3 of their current clients (with enrollment of 6-10 clients total) with respect to enrolling them in the study (per the already approved recruitment methods). Thus, the only revision to this method with respect to recruiting participants to use A4i is a focus on recruitment through a selected group of case managers in addition to the existing broad approach through any case manager.

In the case of clinician outreach, we will orient participating case managers (Using Appendix 5) to the app so they can approach clients who meet the above criteria directly about using a mobile health application, during a regular meeting. In terms of their own participation as study participants, case managers will provide written and informed consent regarding their involvement in the feedback process.

If a client is interested in participating, the case manager may take one of two courses of action, depending on how much available time they feel they have:

1. **No direct clinician involvement for onboarding and installation**: After determining if a client may be interested (using script in Appendix 6), refer the client to the study RA and coordinate a meeting with the client and RA following the next client appointment. During this meeting, the client will be consented in, and follow the procedure as described below (excluding the SCID if the client has a confirmed diagnosis via the case manager). Participating clinicians and A4i client participants who agree as per the revised consent will have discussions about the use and utility of A4i during clinical contacts (see Appendix 7 for suggested questions) while the client remains in the study.

1. **Some direct clinician involvement for onboarding and installation**: After determining if a client may be interested, coordinate with the study RA to consent the client into the trial and follow the below procedures (excluding the SCID if client has a confirmed diagnosis via the case manager) before the next appointment, and *then have the case manager install the application themselves, with the A4i RA present as support*, as a proxy for real-world use of the program. Participating clinicians and A4i client participants who agree as per the revised consent will have discussions about the use and utility of A4i during clinical contacts (see Appendix 7 for suggested questions) while the client remains in the study.

Participating case managers will, subsequent to the A4i pilot, engage in a brief qualitative interview in person, by phone or by REDCap regarding their experience with A4i in setup and in clinical contacts (see Appendix 8 for questions). This qualitative material will be audio recorded in a phone or in person interview, and will undergo content analysis via the Hsieh et al method^27^.

The second strategy also intends to ***explore the potential for ‘real world’ implementation by examining how remote intake and onboarding might work for this application*** in this population.

Self-referred potential participants would be contacted by the RA to go through the same pre-screening as used for the other recruitment strategies used in this study.

After remote consent in (digital form signature) via REDCap or by mail-and-return consent, participant diagnosis will be confirmed through self report of diagnosis and through an affirmative response to one or more of the 4 psychosis items from the BASIS-R^28^. If not a yes, but schizophrenia spectrum is still indicated, the case will be reviewed with the PI (e.g. if the person notes having only negative symptoms, disagrees with chart diagnosis). BASIS-R and BARS questions will be asked by phone interview, which will be audio recorded. The participants will then complete digital survey versions of each of the below surveys by themselves (Basic demographics, BSI, PROM, SFS, cell phone use, others, see below, via REDCap) after which participants will be provided with a link to download the application from a secure download site operated by Memotext, and will be on a call with the RA to troubleshoot installation and get an in-person walkthrough of how to use the app.

The remainder of the trial will run as per the below protocol, with completion occurring 30 days after intake and consenting in. At this time, participants will once again complete mental health measures and other questionnaires as described below online using REDCap. Participants will be asked to report their data usage for the app, and to uninstall the app from their phones at this time. Participants will be unregistered from the provider portal, and will be sent an app resource sheet by email.

Participants will be eligible for a gift card/visa card for up to $85, the final sum of which will be dependent on the degree of their completion of the protocol (divided as described below). Participants will be eligible for $25 in compensation for cellular data overage if they use more data than their current plan allows during the trial.

1. With respect to procedures, in the baseline meeting (in-person 2-3 hours, online, 30min on the phone, the remainder completed online 1.5-2hours) the following would take place:

1. Written consent obtained and capacity to consent confirmed.
2. Diagnosis confirmed via the schizophrenia spectrum module of the Structured Clinical Interview for DSM-5 (SCID-5)^26^ (in person) or using 4 psychosis related elements of the BASIS-R^28^ over the phone for remote participants
3. Basic demographics recorded (age, gender, ethnicity, level of education, age of first illness onset)
4. Completion of the Brief Symptom Inventory (BSI^17^) which will assist in describing the study group and the considerations of the implications of A4i for symptoms.
5. Completion of the Personal Recovery Outcome Measure (PROM^18^) to assess degree of engagement in the recovery process and utilized in the same manner as the BSI.
6. The BARS^19^ will be used to examine implications of A4i for medication use.
7. Social functioning and changes therein will be captured using the Social Functioning Scale (SFS^20^).
8. Client participants will then be asked to indicate mobile technology use indicating quantitatively, in minutes, how long on average they used (i) their mobile device (s) generally, and (ii) proportions of time spent texting, gaming, internet browsing, using the camera, and other purposes and, qualitatively, (i) what they found the most useful/enjoyable in each of these areas and what was less useful/enjoyable.
9. Client participants will be notified both verbally and in the consent form that using A4i will consume some of their cellular data package, and will be offered $25 as compensation for a data top-up to mitigate the chance of data overages.
10. A4i will be uploaded and set up on their phones, either by case managers directly or by the A4i RA. In the case of online set-up, participants will install A4i on their phones themselves, with over-the-phone guidance from the A4i RA.

2. Client participants would take part in 3 weekly check-in calls (10-15 minutes) with the A4i RA to briefly inquire about use in the past week, any challenges, and what seems to be working well. Content from these interactions will be captured through field notes. Research study personnel will send weekly email or text message reminders to participants to confirm time of phone interviews and in-person appointments (Appendix 4). All text messages will be sent from a CAMH approved cell phone or through approved Slaight Centre protocols of email-to-SMS, and will be outgoing only.

3. The app will automatically keep a record of use metrics (total time spent, time spent on specific tools).

At the end of the 1 month period a post evaluation will be completed (60-90 minutes). Aside from demographics and the SCID, this evaluation will include the same measures as those used at baseline in addition to the modified use and utility scale used by Ben-Zeev and colleagues (2014) will be employed to facilitate comparability - itself derived from 3 validated measures of online application use.^21-24^ This will be followed by a semi-structured interview (Appendix 3) addressing use and utility domains as was employed in stage 1. At the end of the meeting A4i will be uninstalled from their phone (they will be provided with a list of evidence based apps from other areas of mental health that address stress, scheduling, and other relevant domains in the event that this experience has prompted an interest in using such tools going forward).

Reimbursement for client participation in the above stage will be $30 for the baseline assessment, $25 for the follow up assessment, and $10 for each of the three phone check ins for a total of $85. Participants will be eligible for $25 as compensation for a data top-up to mitigate the chance of data overages.

Participating case managers will be entered in a draw for a $20 gift card.

Data analysis of stage 2 information will include a descriptive summary statistics for quantitative measures and demographics, a qualitative content analysis to categorize feedback from interviews, a paired sample t-test to examine change in quantitative metrics accompanied by Cohen’s d to examine effect size.

Some Stage 2 participants may be approached to participate in Stage 3 of A4i, and some may be approached to participate in a content creation session at a future date. Content creation would not be research. Participants will be will be told that their participation in these additional activities is not research, is completely voluntary, and will not affect their involvement in future research activities or their health care provision whether they choose to take part or not.

Participants who are approached will be asked if they are interested, and if we can call them to approach them to participate, at which time they will confirm their interest and willingness to participate. Participants will provide verbal consent to this future contact, and the RA will note it on their participant file and electronically in the participant tracking database for the study. The RA will communicate details of the content creation session at a future time when they contact the participant to confirm interest in participating in the session.

Some few participants may be approached to engage as a lived-experience facilitator for these sessions. The RA will obtain verbal consent to approach them by telephone in this capacity in the future, which will be noted on their participant file and electronically in the participant tracking database for the study. The RA will communicate details of the facilitator role with the participant at a future time when they contact the participant to confirm interest in the role.

**Stage 3**

In this third stage of testing we would plan to recruit 30 individuals with a schizophrenia spectrum diagnosis to use A4i for a three week period (15 each for Android and for iOS operating systems). Thirty participants will allow sufficient power for quantitative analyses and will be more than adequate to saturate themes with respect to use and utility.

Eligibility criteria therefore change to:

- Are 18 years of age or older
- Have a schizophrenia spectrum diagnosis
- Own and regularly use a smart phone equipped with an Android or an iOS (Apple) operating system and a talk and data plan, or regular access to wifi
- Read and speak conversational English

Additionally, compensation will be rolled into a single payment for completion of $100 consisting of: $30 for the intake meeting, $10 per phone call (2 phone calls total, one at the end of week one, one at the end of week 2), $25 for completing the study/exit meeting, and $25 for phone using during this time to cover any data overages participants may experience.

All other remaining recruitment, consent, data collection, remuneration for participation, and data analyses remain the same as for Stage 2 of the study.

**Data Management and Integrity**

The basic protection against risk in this study will be provided by Dr. Sean Kidd (study PI). The PI will have primary responsibility for monitoring of participants during the entire time they participate in the study. The PI will meet regularly with study personnel to review accrued data, data confidentiality, and adherence to protocol design, recruitment, and participant complaints. During meetings the Study PI will also review the enrollment data, the accrual and integrity of clinical data, and any adverse event associated with the various components of the study. If a serious adverse event occurs during the study, it will be reported to REB.

All data pertaining to a participant’s involvement in this study will be coded and stored in locked offices. This information will only be accessible to the research team. In unusual cases, a participant’s research records may be released in response to a court order. If the research team learns that a participant or someone with whom the participant is involved with is in serious danger or harm, an investigator will inform the appropriate agencies as per legal or regulatory requirements.

The hard data are stored in a locked filing cabinet stored in a locked office to further protect participant anonymity. Data auditing, entry and quality control will be carried out regularly. Regularly scheduled, and as needed, communications between the study team and the Study PI will clarify any inconsistencies and ambiguities in the data.

At point-of-entry, study data values will undergo consistency edits (e.g., ID validation, range verification, duplicate detection) and personnel will be required to correct errors. Data management staff will run logic error programs to check for accuracy and irregularities within and across data structures and within and across sites. Quality assurance checks will be conducted daily and weekly by site personnel, as well as by data management staff.

**Recruitment, Consent, Risk and Risk Mitigation**

The treating physician/clinical care team will not obtain consent.

A physician investigator or trained research personnel will obtain informed consent. Subjects will be provided with a clear explanation of the objectives, procedures, risks and benefits of the study and all questions will be answered. Questions will be asked of subjects to ensure that they understand the nature of the research, the risks and potential benefits of study participation, and their rights as research subjects prior to obtaining their signature on the informed consent document. Because we believe that consent is an ongoing process in any study, we will continue to educate subjects about the nature of the research and address any questions that may arise throughout the course of the study. We are not planning to use proxy consent.

Participants who are referred from the Slaight Centre for Youth in Transition (SCFYT), will be recruited and pre-screened with the REB approved “pre-screen form” that is implemented across all Slaight studies. At SFCYT, case review meetings are conducted twice a week to review recruitment and to identify new patients who may be eligible for research studies. With their assent, research staff will then meet with potential participants, describe the study in detail, and obtain consent.

Following provision of written informed consent, all participants will be assessed for suitability for inclusion in the study based on the inclusion and exclusion criteria. If it is deemed necessary, research staff may review participants’ CAMH medical charts to obtain additional information to confirm their eligibility or to obtain clinically relevant information for research purposes.

Additionally, as part of the CLEARR (Clinical Engagement and Research Recruitment)

initiative, a delegated Research Coordinator under the supervision of a CLEARR team recruiting clinician and accountable to the Research Manager will identify potential participants and notify the research team and the participant’s clinician about their eligibility to participate in the study. The clinician will then ask their client if they would be willing to meet with a study team member about participating. Only with clients’ agreement will they be approached.

The delegated Research Coordinator will access personal health information (PHI) in I-CARE in order to determine eligibility of CAMH clients to participate in the research study. No PHI except MRN will be collected for this purpose and this information will be kept in a secure locked location per Research Standard Operating Procedures SOP# CR501.

All clients will be given the option to participate. Participation in the study is voluntary. The decision to participate will not affect patients’ receipt of treatment or clinical services. Participants will be informed that they have the option of terminating their participation at any time, without consequence and that no new data will be collected on them. Any existing data will be anonymized.

***Termination of Study:***

Reasons for withdrawing individual participants from the study may include one or more of the following:

1. Major protocol violation
2. Participant lost to follow-up

c) Withdrawal of consent

Any participant may be discontinued from the study at the discretion of the investigators if this is deemed to be in the best interest of the participant. The decision may be made either to protect the participant’s health and safety, or because it is part of the research plan that people who develop certain conditions may not continue to participate.

***Confidentiality***

There is a potential risk of breach of confidentiality that is inherent in all research protocols. Breach of confidentiality will be minimized by the staff who will maintain research data (identified only by participant code number not related to name, or date of birth) in separate charts and a dedicated password protected electronic database. A list of participant names, their ID numbers, and information about how they can be reached will be kept in a separate locked cabinet with access only to study personnel authorized by the PI. Procedures have been established, and will be followed, to minimize the risk of breach of confidentiality. Procedures to maintain confidentially include: (1) formal training sessions for all research staff emphasizing the importance of confidentiality; (2) specific procedures developed to protect participants’ confidentiality, and (3) formal mechanisms limiting access to information that can link data to individual participants. All information obtained from participants will be kept as confidential as possible. Computer based files/data will be entered into password-secured databases and paper-based files will be stored in a secure location. These data will only be accessible to personnel involved in the study and they will abide by confidentiality regulations of the REB. The ethics committee will be granted direct access to the study participants’ original medical records for verification of clinical trial procedures and/or data, without violating the confidentiality of the participants, to the extent permitted by the law and regulations.

In line with CAMH policy, no identifiable information will be saved on the study cell phone and no personal health information will be communicated via text message. All text message communications will be documented by study personnel in the participant case report form.

Research data gathered as part of this study may be shared and provided to other investigators affiliated with the Slaight Family Centre for Youth in Transition (SFCYT) at CAMH for the purpose of data sharing. If participants are enrolled in multiple studies, their research data will be shared across studies to reduce participant burden and avoid duplication of procedures. Only investigators/research team affiliated with SFCYT Centre will have access to secured files and/or research data and will be well-informed regarding the protection of participants’ rights to confidentiality.

Furthermore, investigators collaborating with SFCYT Centre (or other secondary investigators) will have access to the research data collected during the study for the purposes conducting secondary analyses about mental illnesses, such as autism spectrum disorder, depressive disorders, psychotic disorders, bipolar disorders, anxiety disorders, sleep disorders, substance use disorders or dementia (e.g. Alzheimer’s disease). This data will be anonymized and not contain any PHI.

Participants will not be identified by name in any publication of research results. Results will be published as group data without the use of characteristics that would identify individual participants.

*Next Steps*

Based upon feasibility and outcome data generated in Stage 2 another revision will be done with A4i. The next iteration will in turn be employed in a randomized trial which will be the subject of a subsequent protocol.

Findings will also be published in a peer reviewed journal and presented locally.

**References**

1. Alvarez-Jimenez, M., Priede, A., Hetrick, S. E., Bendall, S., Killackey, E., Parker, A. G., ... & Gleeson, J. F. (2012). Risk factors for relapse following treatment for first episode psychosis: a systematic review and meta-analysis of longitudinal studies. *Schizophrenia research*, *139*, 116-128.
2. Yanos, P. T., Roe, D., Markus, K., & Lysaker, P. H. (2015). Pathways between internalized stigma and outcomes related to recovery in schizophrenia spectrum disorders. *Psychiatric Services* *59,* 1437-1442.
3. Harvey, C. A., Jeffreys, S. E., McNaught, A. S., Blizard, R. A., & King, M. B. (2007). The Camden schizophrenia surveys III: five-year outcome of a sample of individuals from a prevalence survey and the importance of social relationships. *International Journal of Social Psychiatry*, *53*, 340-356.
4. Kreyenbuhl, J., Nossel, I. R., & Dixon, L. B. (2009). Disengagement from mental health treatment among individuals with schizophrenia and strategies for facilitating connections to care: a review of the literature. *Schizophrenia bulletin*, *35*(4), 696-703.
5. Kidd, S.A., Virdee, G., McKenzie, K.J. (2014). Mental health reform at a systems level: Widening the lens on recovery oriented care. *Canadian Journal of Psychiatry, 59,*243-249.
6. Goaree, R., Farahati, F., Burke, N., Blackhouse, G., O’Reilly, D., Pyne, J., & Tarride, J. (2005). The economic burden of schizophrenia in Canada in 2004. *Current Medical Research and Opinion, 21,* 2017-2028.
7. Chambliss, C., & Kontostathis, A. (2014). Using mobile technology to enhance psychotherapy for treatment of schizophrenia: a feasibility study. *International Journal of Virtual Worlds and Human Computer Interaction, 2*, 8-17.
8. Seko, Y., Kidd, S., Wiljer, D., & McKenzie, K. (2014). Youth mental health interventions via mobile phones: a scoping review. *Cyberpsychology, Behavior, and Social Networking*, *17*, 591-602.

Ben-Zeev D, Kaiser, SM, Brenner CJ, Begale M, Duffecy J, Mohr DC. (2013). Development and usability testing of FOCUS: a smartphone system for self-management of schizophrenia. Psychiatric Rehabilitation Journal. DOI: 10.1037/prj0000019

1. Palmier-Claus, J. E., Ainsworth, J., Machin, M., Barrowclough, C., Dunn, G., Barkus, E., ... & Salter, E. (2012). The feasibility and validity of ambulatory self-report of psychotic symptoms using a smartphone software application. *BMC psychiatry*, *12*, 1.

Miller BJ, Stewart A, Schrimsher J, Peeples D, Buckley PF. (2015). How connected are people with schizophrenia? Cell phone, computer, email and social media use. *Psychiatry Research, 225*, 458-463

1. Hargreaves, A., Dillon, R., Anderson-Schmidt, H., Corvin, A., Fitzmaurice, B., Castorina, M., ... & Donohoe, G. (2015). Computerised working-memory focused cognitive remediation therapy for psychosis—A preliminary study. *Schizophrenia research*, *169*, 135-140.
2. Ben-Zeev, D., Brenner, C.J., Begale, M., Duffecy, J., Mohr, D.C., & Mueser, K.T. (2014). Feasibility, Acceptability, and Preliminary Efficacy of a Smartphone Intervention for Schizophrenia. *Schizophrenia Bulletin, 40,* 1244-1253.
3. Kane, J. M., Robinson, D. G., Schooler, N. R., Mueser, K. T., Penn, D. L., Rosenheck, R. A., ... & Marcy, P. (2015). Comprehensive versus usual community care for first-episode psychosis: 2-year outcomes from the NIMH RAISE Early Treatment Program. *American Journal of Psychiatry*.
4. Kidd, S. A., Herman, Y., Barbic, S., Ganguli, R., George, T. P., Hassan, S., Maples, N. & Velligan, D. (2014). Testing a modification of cognitive adaptation training: streamlining the model for broader implementation. *Schizophrenia Research*, *156*(1), 46-50.
5. Boland, M. V., Chang, D. S., Frazier, T., Plyler, R., Jefferys, J. L., & Friedman, D. S. (2014). Automated telecommunication-based reminders and adherence with once-daily glaucoma medication dosing: the automated dosing reminder study. *JAMA Ophthalmology*, *132*(7), 845-850.
6. Derogatis, L., & Melisaratos, N. (1983). The Brief Symptom Inventory: An introductory report. *Psychological Medicine,* *13*(3), 595-605. doi:10.1017/S0033291700048017
7. Barbic, S., Kidd, SA, Backman, C.,MacEwan, W.. Honer, W., & McKenzie, K. (2015). How can Rasch measurement methods inform a paradigm shift in mental health care? *Quality of Life Research, 24,* 76.
8. Byerly MJ, Nakonezny PA, Rush AJ. (2008). The Brief Adherence Rating Scale (BARS) validated against electronic monitoring in assessing the antipsychotic medication adherence of outpatients with schizophrenia and schizoaffective disorder. *Schizophr Research, 100,* 60–69.
9. Birchwood M, Smith J, Cochrane R, Wetton S, Copestake S. (1990). The social functioning scale: The development and validation of a new scale of social adjustment for use in family intervention programmes with schizophrenic patients. *The British Journal of Psychiatry, 157*, 853-9
10. Brooke J. (1996). SUS-A quick and dirty usability scale. *Usability Evaluation in Industry.* 189–194.
11. Lewis JR. (1992). Psychometric evaluation of the poststudy system usability questionnaire: the PSSUQ. In: Proceedings of the Human Factor Society Annual Meeting. 1992;36:1259.
12. Venkatech V, Davis FD. (2000). A theoretical extension of the technology acceptance model: four longitudinal field studies. *Management Science, 46,* 186–204.
13. Lund AM. (2001). Measuring usability with the USE questionnaire. *Usability and User Experience, 8,* 8.
14. Sheehan, D., Lecrubier, Y., Sheehan, K., Amorim, P., Janavs, J., Weiller, E., ... & Dunbar, G. (1998). The Mini-International Neuropsychiatric Interview (MINI): The development and validation of a Structured Diagnostic Psychiatric Interview for DSM-IV and ICD-10. *The Journal of Clinical Psychiatry. Supplement*, *59*(20), 22-33.
15. First MB, William JBW, Karg RS, Spitzer RL: Structured Clinical Interview for DSM-5-Research Version (SCID-5 for DSM-5, Research Version; SCRID-5-RV). Arlington,VA, American Psychiatric-Association, 2015.
16. Hsiu-Fang Hsieh & Sarah E. Shannon. (2005). Three Approaches to Qualitative Content Analysis. Qualitative Health Research. Vol 15, Issue 9, pp. 1277 – 1288. DOI:10.1177/1049732305276687
17. 28. Eisen SV, Normand S-L, Belanger AJ, Spiro A, Esch D. (2004). The revised Behavior and Symptom Identification Scale (BASIS-R) reliability and validity. Med Care. 42(12):1230-1241

**Appendix 1 – Stage 1 Case Manager Script**

I’d like to tell you about a project that is happening that involves some early testing of a cellphone application designed to assist people with schizophrenia in a range of areas – focusing on improving coping and improving social engagement. It would involve installing the app on your phone, you using it every day for a week and completing some surveys about how useful it is and about where you are at in terms of day to day functioning. You would get paid for your involvement. It is completely fine to say no to this – doing that will not affect the services you receive in any way. If you are interested, I would pass your name and phone number along to the research assistant for the project who would tell you more about it. No need to decide right now. Just let me know when you like if you are interested.

**Appendix 2 – Recruitment Flyer**

**Please see appended file.**

**Appendix 3 – Use and Utility Interview for the A4i RA**

1. What is your overall impression of A4i so far?
2. What functions have been helpful/unhelpful? Why? (if not generated prompt responses for each key function)
3. If you could change some things about A4i what would you change? Why?
4. (if not already addressed) What impacts does using A4i have in your life if any? How does it have those impacts?
5. Did you have any recurring software errors while you were using A4i? What were they?
6. Any other thoughts?

**Appendix 4 – Text message reminders**

“Hi this is A4i! A quick reminder that our appointment is for tomorrow at **:**AM/PM. See you then!”

Or

“Hi this is A4i! A quick reminder that our appointment is for tomorrow at **:**AM/PM. We will call you then!”

**Appendix 5 –Case Managers Info Sheet – for non-remote recruitment**

There is an opportunity for your clients to participate in a trial testing a mobile mental health application beta called App for Independence. This app is designed for use by people with a diagnosis in the schizophrenia spectrum, who speak and read English, are 18 years of age or older, who own and use an Android or iPhone smartphone with a data and talk plan, and can travel to downtown Toronto twice to have meetings before and after trying out the application.

Using the app is a way to keep track of appointments and important activities, provide participants with helpful information and coping strategies for their mental health, help participants check if voices they hear in the environment are real or not, allows participants to share stuff with other users of the app in an anonymous and moderated manner, and enables participants to keep track of their day-to-day health through a daily check in. This app is also for people who do not have problems with using technology, or who are worried about having the A4i software track what they enter into the program during the trial.

Participant involvement would include two trips to the Centre for Addiction and Mental Health downtown Toronto, to participate in a consenting-in process, to complete some questionnaires about their mental health, to have the mobile application installed on their phone, and go through a quick tutorial to help them use the application.

After this initial appointment, the trial would last for 30 days (Stage 2)/ three weeks (Stage 3). The study RA would call the participant once a week, on week days only, at their convenience during the month (Stage 2)/ three weeks (Stage 3) they are using the app to follow up with questions about how they are enjoying its use.

Case managers may ask the participants set questions about use and utility of A4i at regular meetings.

Participants may get email or text message reminders during this time to remind them about phone appointments and the final meeting but this is something they can agree to or not depending on how they feel about it.

At the end of the 30 day (Stage 2) /three week(Stage 3) trial, the participant will return to the 252 College Street to complete some questionnaires, have the mobile application removed from their phone, and be given a resource sheet.

All of the information provided and participation in the trial will remain confidential and private. Participating in this trial or not will in no way affect their current care.

Participants will be compensated for their participation up to a total of $85 (Stage 2)/$100 (Stage 3), and (for Stage 2 but not Stage 3) would also be eligible for $25 of compensation for any data overage they might incur while using the app.

**Appendix 6 – Stage 2/3 Case Manager Script**

I’d like to tell you about a project that is happening that involves some early testing of a cellphone application designed to assist people with schizophrenia in a range of areas – focusing on improving coping and improving social engagement. It would involve installing the app on your phone, you using it for a month(Stage 2) / three weeks (Stage 3) and completing some surveys about how useful it is and about where you are at in terms of day to day functioning. You would get paid for your involvement. It is completely fine to say no to this – doing that will not affect the services you receive in any way. If you are interested, I would pass your name and phone number along to the research assistant for the project who would tell you more about it. No need to decide right now. Just let me know when you like if you are interested.

**Appendix 7 – Use and Utility Interview for Clients delivered by Case Managers**

1. Do you like using A4i? Do you find it useful?
2. What functions have been helpful/unhelpful? Why? If not generated, prompt responses for each key function:
   1. medication, social, or appointment reminders
   2. social participation via contributing to the feed
   3. voice checker
   4. toolkit and psychoeducation
3. If you could change some things about A4i what would you change? Why?
4. What impacts does using A4i have in your life if any? How does it have those impacts?
5. Any other thoughts about A4i?

**Appendix 8 - Stage 2 Case Manager Questionnaire for use and utility of A4i**

Each question to be answered on a 5 point Likert scale, or by filling in a blank.

Post-installation questions:

1. How easy was explaining to your client what A4i does?
   1. If you ran into difficulties, what were they?
2. How easy was the installation of the A4i app during your client meeting? If you ran into difficulties, what were they?
3. How long did installation and explanation of the app to your client take?
4. Did your client have many questions about the app at the time of installation?

Post-use questions:

1. Do you feel that using A4i improved your client’s quality of life?
   1. If not, why not?
2. Do you feel that using A4i improved your client’s medication adherence?
   1. If not, why not?
3. Do you feel that using A4i improved your client’s social engagement?
   1. If not, why not?
4. Do you feel that using A4i improved your client’s psychosocial education level?
   1. If not, why not?
5. Do you feel that using A4i improved your client’s level of insight into their disease?
6. What further improvements could be made to A4i to make it fit better with your client population?
7. What were the major complaints about the app?
8. What are your major complaints about clients using the app?
